# Cosmovision as Cognitive Technology: The Case of Mesoamerican Medicinal Knowledge

**DOI:** 10.1111/tops.70008

**Published:** 2025-04-28

**Authors:** Johan De Smedt, Helen De Cruz

**Affiliations:** ^1^ Department of Philosophy Saint Louis University

**Keywords:** Cognitive technologies, Affordances, Indigenous medicinal knowledge, Nahua (Aztec), Cruz‐Badianus codex

## Abstract

We examine the use of cognitive technologies in the acquisition and retention of botanical and medicinal knowledge. We focus on the Cruz‐Badianus codex, a 16th‐century Nahua (Aztec) herbarium which discusses the use of plants for a range of illnesses. We show how the codex reflects the Mesoamerican cosmovision, in particular, the association of the human body and cosmos, and the polarity and balance of hot and cold. We hypothesize that the cosmological and philosophical ideas that underlie the medicinal uses prescribed in the codex are not incidental, but rather help to scaffold knowledge, retain in memory successful remedies, and aid the transmission of information.

## Abstract 2

We examine the use of cognitive technologies in botanical knowledge for medicinal purposes in the Nahua (Aztec) Cruz‐Badianus codex. The Mesoamerican cosmovision, especially the association of human body and cosmos, is reflected in the text.

## 1 Introduction

In May 2024, a male Sumatran orangutan healed himself with a medicinal plant. The animal was injured in the face, likely following a fight with a conspecific. He chewed leaves of the liana *Fibraurea tinctoria* and fully covered his wound with the resulting paste. This vine is known in traditional medicine for its anti‐inflammatory and analgesic properties. A month later, his wound was healed (Laumer et al., 2024). This behavior fits a pattern of self‐medication in nonhuman animals. It is widely attested among different taxa, not just great apes such as chimpanzees (Freymann, Carvalho, Garbe, Ghazhelia, & Hobaiter, 2024) and gorillas (Yinda, Onanga, Obiang, Begouabe, & Akomo‐Okoue, 2024), but also birds that use twigs of parasite‐repelling plants when building nests (Mansouri et al., 2021) and even ants (Frank et al., 2024). Self‐medication is likely transmitted through various forms of social learning (e.g., Whiten, Goodall, McGrew, Nishida, & Reynolds, 1999; Schuppli & van Schaik, 2019).

While humans are but one species that uses plants for medicinal purposes, our botanical knowledge exceeds that of other animals in scope and detail, with sophisticated taxonomic systems of plants to treat a variety of ailments, including psychological conditions, problems in childbirth, and infectious diseases (see, e.g., the papers collected in Medin & Atran, 1999; Ragupathy & Newmaster, 2009; Cardoso, de Queiroz, Bandeira, & Góes‐Neto, 2010). We can do this because our external memory systems complement and extend internal mental representations (Donald, 1991). We have used cognitive technologies such as maps, tallies, visual imagery of animals, and calendars since at least the Late Paleolithic (De Smedt & De Cruz, 2011; Bacon et al., 2023). Cognitive technologies also include forms that we might not ordinarily think of as “external,” such as songs or recitation techniques that help to memorize vast amounts of knowledge. For example, songs by Warlpiri (Indigenous Australian) women help to transmit topographic information about the landscape by embedding it in a deep present “dreaming” through stories about ancestors (Barwick, 2023). Young boys on the Micronesian atoll of Polowat traditionally learned to navigate by a star compass. This is a chart where setting and rising stars are used as visual anchors for the location of specific islands. Before they board their first canoe as navigator, they must flawlessly rattle off the names and positions of stars and islands, the star compass only present in their head (Gladwin, 1970).

The wide diversity of cognitive technologies leads us to wonder how they can become fitting vehicles that transmit knowledge externally. What kinds of facilitation take place, which sorts of affordances do they allow? Our current cultural context where vast amounts of information is stored on paper and in electronic format may lead us to think that the format is not relevant, but that only the content counts. It seems irrelevant whether data (say, the collected works of William Shakespeare) are stored in audio format, microfilm, or in the cloud. Moreover, we are heirs to an Enlightenment ideal of universal human cognition. This ideal was helpful to challenge racist assumptions about “primitive minds”: 2+2 will always be 4, and 180° is always the total sum of the angles of a triangle, no matter who considers these propositions (see Bender & Beller, 2011 for discussion). However, these assumptions of culture‐ and medium‐independence obscure the fact that our culture‐specific access to cognitive technologies has a deep influence on what kinds of knowledge we can discover and transmit. The format of storage, far from being irrelevant, has deep implications for how well something will be transmitted and how it will be retained in individual memory. For this reason, anthropological and historical studies are indispensable in the study of cognition and culture.

In this paper, we examine how the Mesoamerican cosmovision—in particular, the view of a close connection between the human body and the cosmos, and the resulting cold‐hot humoral system—facilitated and still facilitates the transmission of knowledge of plants for medicinal purposes. It does so by posing an intimate link between the human body, human societies, and the natural world, thus creating affordances that help people look for plants in their natural environment to cure various ailments. As originally defined by Gibson (1979), affordances are features of the environment that offer an agent ways to interact with it: for example, a couch invites people to slouch (i.e., a couch *creates the affordance* of slouching), whereas a chair indicates more upright postures. As a result, people will tend to sit differently on couches compared to chairs. Gibson originally had in mind features of the environment that are purely physical. However, affordances can also be created through knowledge of the environment. For example, a type of nut could be poisonous (not‐eatable), but knowledge of how to roast the nut to release its toxins could create a different affordance: the roasted nut is for‐eating. Similarly, we will see how cognitive technologies of medical knowledge create affordances of using plants for specific pharmaceutical uses.

Section 2 provides a brief overview of core biological knowledge and the Mesoamerican cosmovision. Section 3 examines the Cruz‐Badianus codex from 1552 as an exemplification of these principles. This document uses the western format of a herbarium to showcase Indigenous Nahua knowledge of medicinal plants. Section 4 shows how this Indigenous medical knowledge had an influence on western medicine in the 16th and 17th centuries and describes how this fits in a continued pattern of use of Indigenous knowledge in pharmaceutical contexts. Section 5 offers some suggestions on how to decolonize Indigenous pharmacological knowledge.

## 2 Core knowledge and the Mesoamerican cosmovision

Across cultures, humans parse the world in different ontological domains, termed “core knowledge” or “intuitive ontologies” (e.g., Spelke & Kinzler, 2007, De Cruz & De Smedt, 2007). These are sets of expectations about the world which emerge early in development, and which represent salient aspects of our lives that come with distinct and specialized inference mechanisms. For example, young infants believe that physical objects continue to exist when they are occluded (Baillargeon, 1987), they estimate the number of objects in a set (Izard, Dehaene‐Lambertz, & Dehaene, 2008), and they expect that agents behave according to internal mental states (Kovács, Téglás, & Endress, 2010). These beliefs correspond to core knowledge of physics, numerosity, and theory of mind, respectively.

The natural world is an essential part of our lives. Knowledge of plants and animals helps us assess opportunities and risks, for instance, which plants are edible, and which ones are poisonous. Mistakes could be fatal. Thus, intuitive biology is plausibly a domain of core knowledge, about which we have sets of early‐developed ontological expectations. Evidence for this comes from cross‐cultural similarities in folk taxonomies. Across cultures, humans divide the living world into hierarchical taxonomies. These include a basic level of folk species (e.g., dog, starling, oak tree) as well as lower and higher levels such as kingdoms (plant, animal) and specific varieties (e.g., Irish setter, American red oak) as well as ecological groupings, such as carnivores and trees (Atran, 1999). This universal taxonomy results in striking similarities at the species level between scientific and Indigenous systems (e.g., Diamond & Bishop, 1999). Moreover, Cardoso et al. (2010) found that folk classifications of fungi among several Indigenous Brazilian groups, including the Caiabi, Txicão, and Yanomami, reflect deep phylogenetic relationships: the Yanomami have classification schemes that are similar to recently proposed scientific phylogenetic trees.

These remarkable similarities hint at universal cognitive constraints on how we think about the natural world. However, universal constraints do not mean that cultural particulars are irrelevant. To the contrary, culturally variable cognitive technologies can give us deeper insights into how culture and cognitive biases operate together to facilitate our understanding of the natural world. Here, we take the Mesoamerican cosmovision as a case study to show how cultural scripts can create unique engagements with the environment. While our paper is focused on Nahua medicine and Mesoamerican cosmovision, it is worth pointing out that cosmovisions outside of this cultural sphere also shape views about health and the environment. To give but one brief example, Qollahuaya Andeans live on Mount Kaata (midwestern Bolivia). They live in three separate villages that occupy three ecological levels of the mountain at altitudes between 3.2 and 4.3 km. These communities are united by the exchange of resources and spouses. They grow different foods (potatoes, oca, barley) and animals (alpaca, sheep, pigs) they exchange with those that live at other ecological levels. They conceive of their mountain as an *ayllu*, a vertical territory divided into low, middle, and high ecological zones, with communities that have close historical, social, and cultural bounds settled at each level (Bastien, 1978). They draw an analogy between *ayllu* and its waterways to understand how the human body works (Bastien, 1985). They conceive of bodily fluids (such as blood, fat, and water) as a complex hydraulic system that needs to function well for people to be healthy. The mountain is used as an affordance to think about human physiology and diseases. For example, just like villagers on the mountain exchange animals and plants to eat, body parts exchange bodily fluids and fat; just like underground streams connect the different waterways and lakes on the mountain, arteries transport blood throughout the body. They are skilled herbalists who use more than 1000 medicinal plants in their healing practices that go back to the 8th century. Fellow Andeans throughout Argentina, Bolivia, Perú, and Chile call their itinerant healers *Qolla Kapachayuh*, Lords of the Medicine Bag (Bastien, 1982).

Historical Mesoamerica overlaps significantly with the geographic location of Mexico. This area is a dense patchwork of cultural zones and environmental ranges, including deserts, rainforests, and coastal regions, which gives it a rich botanical diversity. Among its peoples were the Mexica, a Nahuatl‐speaking^1^ group of the Valley of Mexico, a highlands plateau in central Mexico. They were the rulers of the Triple Alliance, better known as the Aztec Empire. The Triple Alliance consisted of three *altepetl* (city states), Tenochtitlan, Texcoco, and Tlacopan. They ruled the Valley of Mexico and the surrounding areas from 1428 until 1521, when they were defeated by the conquistador Hernán Cortés and his allies.

Despite their substantial cultural diversity, several authors (e.g., López Austin, 1996; Robles‐Zamora, 2021; but see Muñoz, 2017 for critique on the terminology) have hypothesized that past and present Mesoamerican cultures hold a common *cosmovision*. A cosmovision is an ontology shared between members of a culture, or related cultures, that situates human beings in the world and that shapes possibilities for action. The Mesoamerican cosmovision is a shared ontology that posits humans as part of a larger cosmic whole, which consists of three closely interrelated aspects: human, natural, and spiritual. These three spheres frequently intermingle, as, for example, in the *Día de los Muertos*, a syncretistic celebration where dead friends and relatives are invited into the sphere of the living and celebrate together. Humans have a kinship relationship to nonhuman persons (plants and animals): the category “person” is extended and includes “other‐than‐human persons,” which incorporate nonhuman animals, plants, and certain minerals and stones (see De Smedt & De Cruz, 2023 for an overview). Furthermore, the human body reflects the cosmos and the natural world, as can be seen in the prominent use of anthropomorphic imagery in Mesoamerican art.

The cosmovision governs morality, which is not restricted to relationships between humans but extends to human−environment relationships, including agriculture and horticultural practices. Agriculture is not a morally neutral activity but involves a duty of care for both wild and domestic plants (Salmón, 2000), such as sustainable sowing and harvest. Binary contrasts between hot‐cold and positive‐negative are an integral element of this moral system (Robles‐Zamora, 2021). Rather than seeing these binary polarities as opposites, they are harmonious constitutives of the world and need to be in balance for our moral and natural environments (which, as we just saw, are not sharply differentiated in the Mesoamerican cosmovision) to be well.

Traditionally, Mesoamericans distinguished between three kinds of souls or animistic forces, *tonalli*, *teyolia*, and *ihíyotl*, which are present in our bodies as well as in meteorological elements. *Tonalli* is situated in the head and is associated with heat, life force, and blood, which carries it around, and is also present in the sun. *Teyolia* sits in the heart and is associated with memory and intelligence, as well as rain and water. *Ihíyotl* resides in the liver; it is the center of our emotions and is associated with wind and air (Gimmel, 2008; Furst, 1995). Human health requires a balance between and within these three forces.

The Mesoamerican cosmovision is transmitted through stories, songs, material culture, and various forms of apprenticeship. Drawing on affordance theory, Alfredo Robles‐Zamora (2021) argues that its conceptual schemas allow Mesoamericans to interact with their environment in specific ways. A cosmovision is not an abstract worldview; it has a relational, practical character. For example, some cosmovisions such as Christianity as it developed in Europe and its colonies posit a fundamental difference between humans and other animals, originating in the view that humans were created in God's image and should have dominion over the rest of nature. This view makes it hard for Christians and their intellectual heirs to pay proper attention to the impact of their activities on the environment (White, 1967; De Cruz & De Smedt, 2022). By contrast, according to Mexican (Rarámuri) Indigenous anthropologist Enrique Salmón,

Indigenous people in North America are aware that life in any environment is viable only when humans view their surroundings as kin; that their mutual roles are essential for their survival. To many traditional Indigenous people, this awareness comes after years of listening to and recalling stories about the land (Salmón, 2000, 1327).

Within this *kincentric ecology*, Mesoamericans see themselves as fundamentally related to all of nature, and see plants and animals as their relatives, the “kinship of plants and people” (Salmón, 2020). Next to understanding the impact of human actions on the environment, this view makes it easier to both conceptualize plants as being useful for health and to have their own ecological relationships. Contemporary Indigenous Mexican populations such as the Rarámuri still practice sustainable agricultural techniques, for example, the controlled burning of oak trees and the rewilding of agricultural fields. This helps the vegetation to regenerate after burnt patches have been used as bean fields and prevents soil depletion and forest fires (LaRochelle & Berkes, 2003). We will now examine how the Mesoamerican cosmovision creates specific affordances for Nahua people to think about the medicinal use of plants, using the Cruz‐Badianus codex as an illustration.

## 3 The Cruz‐Badianus codex

The *Libellus de Medicinalibus Indorum Herbis* is a manuscript originally composed in Nahuatl by a Nahua physician whose Spanish name was Martín de la Cruz. It was translated into Latin by Juan Badiano, a fellow Nahua, who like de la Cruz was an instructor at the Colegio de la Santa Cruz in Tlatelolc. This manuscript is usually referred to as the Cruz‐Badianus codex (or *Codex de la Cruz Badiano* in Spanish); its name in Nahuatl would have been *Amate Cehuatl Xihuitl Pitli*. It is the earliest surviving Mesoamerican Indigenous herbarium. Unfortunately, the original in Nahuatl disappeared, but the Latin 1552 translation remains (de la Cruz, 1552, is an online version of the Latin codex). It features 185 color illustrations of local plants painted by Indigenous illustrators, together with their medicinal uses. It was a fundraising gift for Charles V, then King of Spain, to raise money for and prevent the closure of El Colegio de la Santa Cruz, where young Nahua boys were trained for Roman Catholic priesthood (Chavarría & Espinosa 2019). Unfortunately, the effort failed, and the college fell in ruin soon after. Indigenous people were forbidden to become priests from 1555 onward (Poole, 1981).

**Fig. 1:**
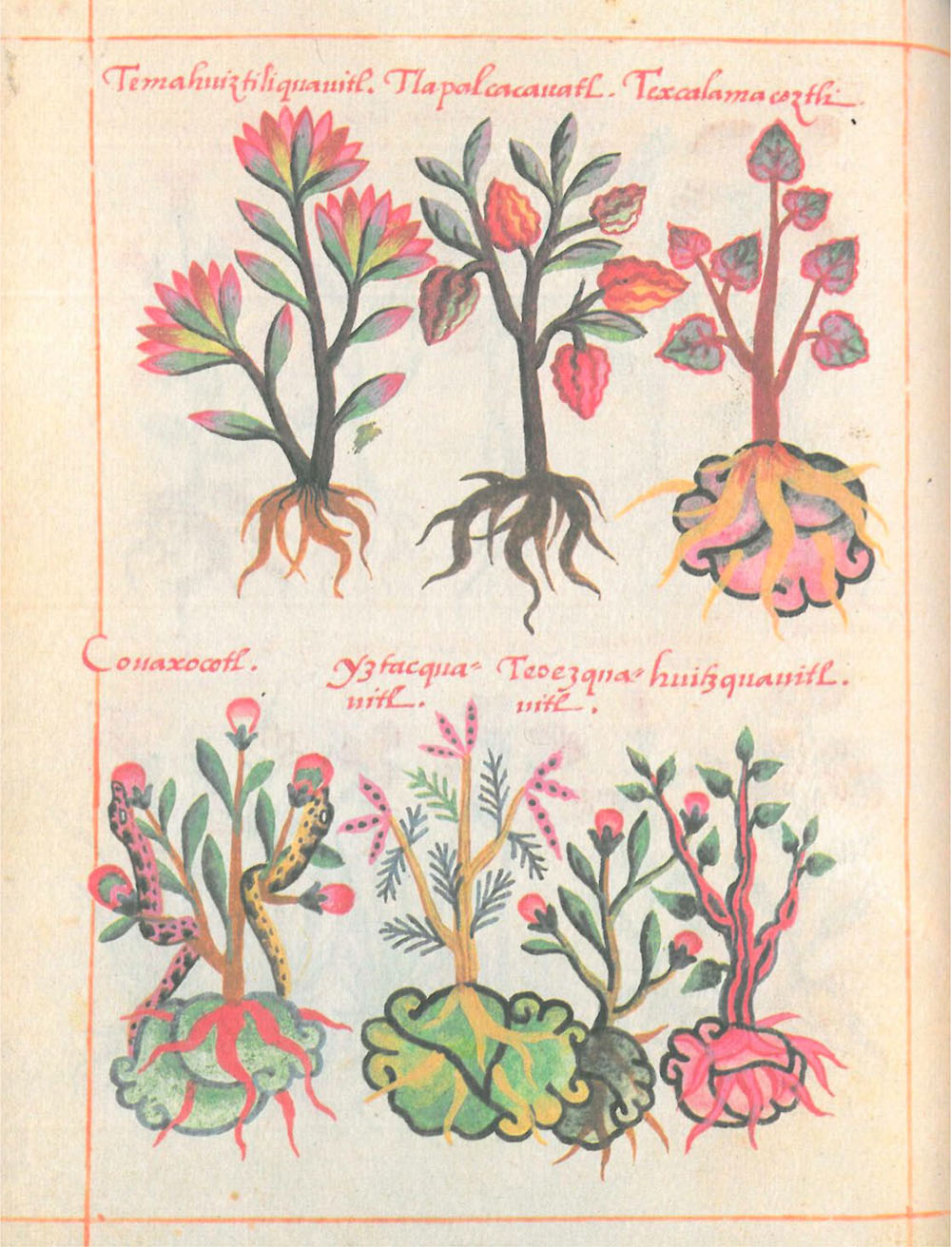
Cruz‐Badianus codex, 38v. All figures in this paper are from the online Wikimedia version of the codex (de la Cruz, 1552).

There has been an ongoing debate on whether the Cruz‐Badianus codex is highly influenced by Western herbaria (e.g., Hassig, 1989) or whether it presents primarily Native insights (e.g., Gimmel, 2008). The current consensus recognizes an influence of both western and Native science (e.g., Poskett, 2022). The organization of plants into an illustrated compendium is clearly based on western herbaria. However, the lush plant illustrations combine the visual vocabulary of western herbaria (for instance, in the use of graded colors and lack of strong outlines) with Nahua imagery. For example, the Nahua stone glyph frequently appears under the roots of plants (e.g., Fig. 1, top, third plant on the right, and all plants on the bottom) which may indicate the type of terrain on which the plants grow. Also common is the use of name glyphs, as in Fig. 1, bottom, left, where we see two snakes slithering up a plant, which is the Nahua name glyph for *couanaxocotl*. This is a contraction of *cohuiatl*, or *coatl* (serpent) and *xocotl* (sour fruit), thus, serpent fruit. The identification is uncertain, perhaps a *Physalis* (Tucker & Janick, 2020). Also, all plants are labeled with their names in Nahuatl (transcribed into the Latin alphabet). Several of the plants illustrated in this codex have not been identified.

**Fig. 2:**
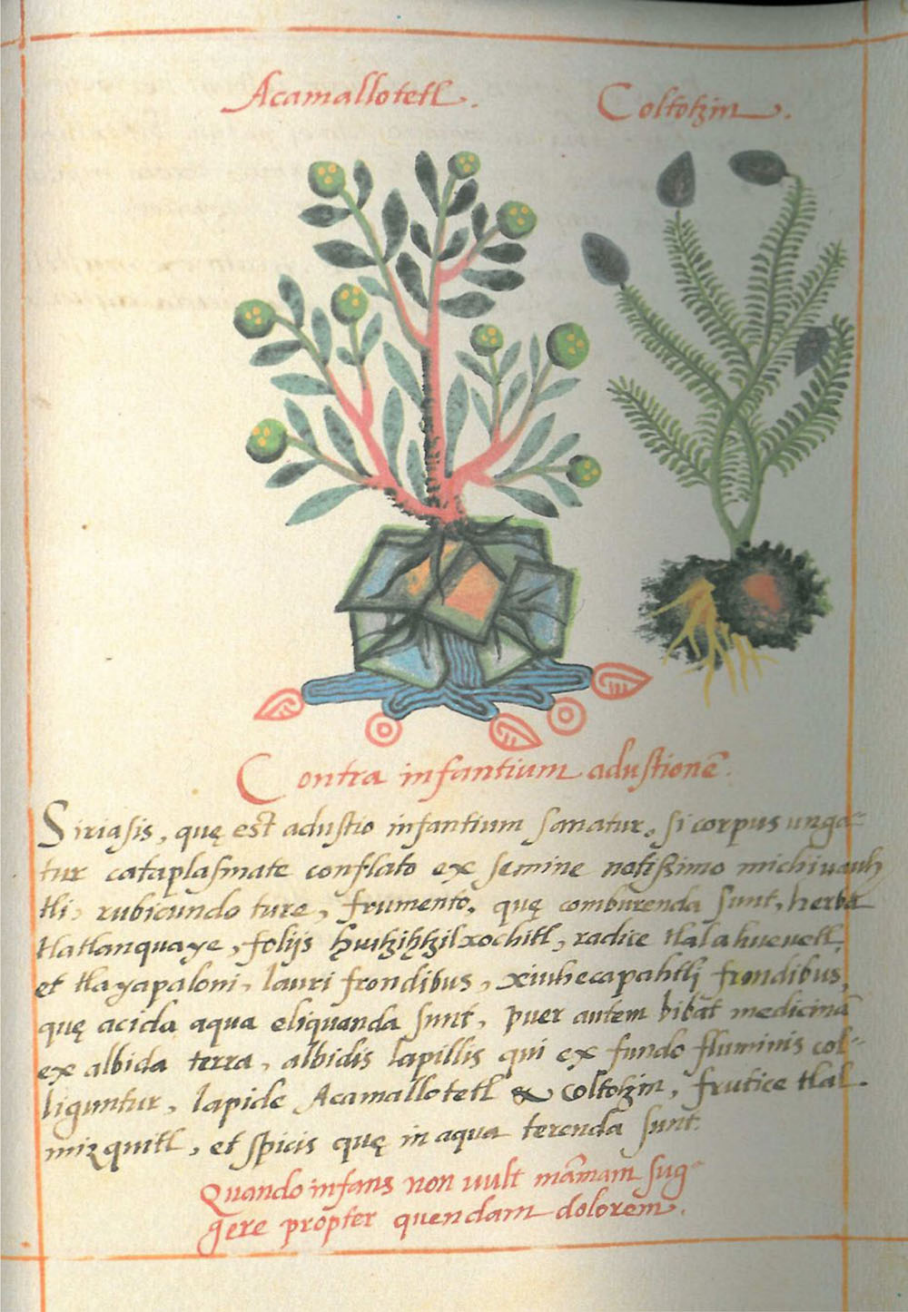
Cruz‐Badianus codex, 61r.

The Nahua glyph for water sits right under the roots of the *acamallotetl*, colored in a deep azure blue (Fig. 2, left). The accompanying text explains the plant helps to stop skin eruptions in infants. It also mentions water: “the small white stones gathered from the bottom in flowing water, the stone *a‐camallo‐tetl* and *coltotzin*… ground in water” (Gates, 1939, 113).^2^

The codex is organized along the different parts of the human body that need treatment, moving from head to toes, treating minor inconveniences such as male hair loss and smelly armpits, as well as major diseases such as cataract and glandular tumors. It also discusses childbirth, how to get infants to breastfeed, what to do if you are struck by lightning, and palliative care for the dying. The relation of the human body to the natural world set out in this book exemplifies the Mesoamerican cosmovision: there is a natural link between the human body and its wellbeing and the plants in our environment. The different treatments in the codex recall the Mesoamerican view of the three animistic forces. For example, the use of flowers in chapter 8 prescribes aromatherapy against lassitude: “One fatigued will be restored if the feet be bathed in choice liquor, with the *ahuiyac‐xihuitl* or *tlatlanquaye*, *tlatlaolton*, *itzcuin‐patli*, *xiuh‐ecapatli*, *iztauh‐yatl*, the *huitzihtzil‐xochitl* flower, and the stones *tetlahuitl*, *tlacal‐huatzin* and *eztetl*, to be crushed in hot water” (Gates, 1939, 66). The smells will help to increase *tonalli* (recall, a concept akin to life force); given that the *tonalli* is associated with heat, the flowers and stones are crushed in hot water. Specifically for people in administrative governmental functions “all fine smelling summer flowers” are used to relieve fatigue (Gates, 1939, 70). This reflects beliefs about societal hierarchy: for the Nahua, members of their high class (who were involved in state administration) had greater amounts of *tonalli* and needed continuous strengthening (Gimmel, 2008).

A key governing principle underlying the codex and the medical principles behind it is the balance between hot and cold. Diseases are conceptualized as an imbalance of cold and hot elements. Medicinal plants as well as interventions such as cold and hot baths help to restore balance to the body. For example, someone who has overheated eyes should wipe their face with “the squeezed juice of the bushes *oco‐xochitl*, *huacal‐xochitl*, *matlal‐xochitl* and *tlaco‐izqui‐xochitl*” (Gates, 1939, 14), which are plants that are associated with cold. Moreover, “one suffering from a defect of the eyes should abstain from sexual acts, the heat of the sun, smoke and wind, not use *chilmolli* [spicy sauce] as a sauce in his food, not eat hot foods. On his neck he must carry a red crystal, and not look at white things but black” (Gates, 1939, 15).

According to some authors, such as George Foster (1987), one of the founders of medical anthropology, this humoral cold‐hot system is a simplified folk variant of classical Greco‐Persian humoral pathology that was imported into Latin America. It was first described in Peru in 1877 (which is a lot later than 16th‐century Mexico). However, it may also be an ancient Amerindian system that was maintained throughout the colonial regimes precisely because of its similarities with the Galenic system. Given the polarity of opposites in the Mesoamerican cosmovision, and the early date of the codex, this hypothesis seems quite likely. Indeed, García‐Hernández et al. (2021, 2023) argue for a cold‐hot system that is widespread in Mesoamerica with several local variations. It is also found throughout Latin America: consider the Qollahuaya. As Bastien (1985, 607) points out, Greek humoral pathology is rooted in philosophical speculation about the nature of the universe, which is composed of four elements (fire, water, earth, and *aer*) that correspond to four bodily fluids, and through these to emotions, personality types, and sickness. The body, in a way, is analogous to the universe. Qollahuaya hydraulic physiology, on the other hand, is rooted in perceived similarities and relationships between human physiology and Mount Kaata. Moreover, the Qollahuayas have a fundamentally different way of understanding nature. Whereas the ancient Greeks understood nature as a system in balance, the former view nature as a cyclical system: the hydraulic cycle of fluids in underground waterways and human arteries, the proper function of which guarantees a good health.

**Fig. 3:**
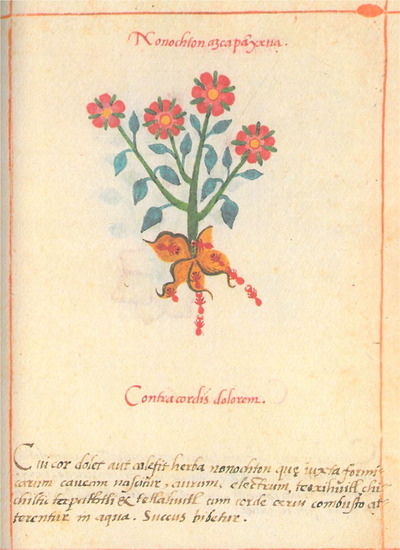
Cruz‐Badianus codex, 28r.

Although the Cruz‐Badianus codex is focused on the healing properties of plants, its iconography also gives some information about how to identify them and in what sort of soil they grow (e.g., rocky or limy).^3^ For example, Fig. 3 shows *nonochton*, a plant used in a liquor against heart pain which is said to grow near red ant nests. While the combination of red flowers and red ants might point to a symbolic connection, this is not necessarily the case. The ethnobotanist Gary Nabhan and colleagues (Tewksbury et al., 1999) were able to confirm a dependence between red species that Native groups living in the sierras of Sonora, Mexico and Arizona, USA have pointed out. Northern cardinals (*Cardinalis cardinalis*) perch and forage preferentially among the canopies of the desert hackberry (*Celtis pallida Torr*.). Among local bird species, they are also the primary dispenser of the wild chile plants (*Capsicum annuum* L. var. *aviculare*). This foraging behavior results in a nonrandom distribution of wild chiles under the dense tree canopies rather than under the desert sun, even though they are deprived of sunlight there. The northern cardinal is a red bird, the ripe fruits of both desert hackberry and wild chile are red, but the dependence between them is not symbolic but ecological.

**Fig. 4:**
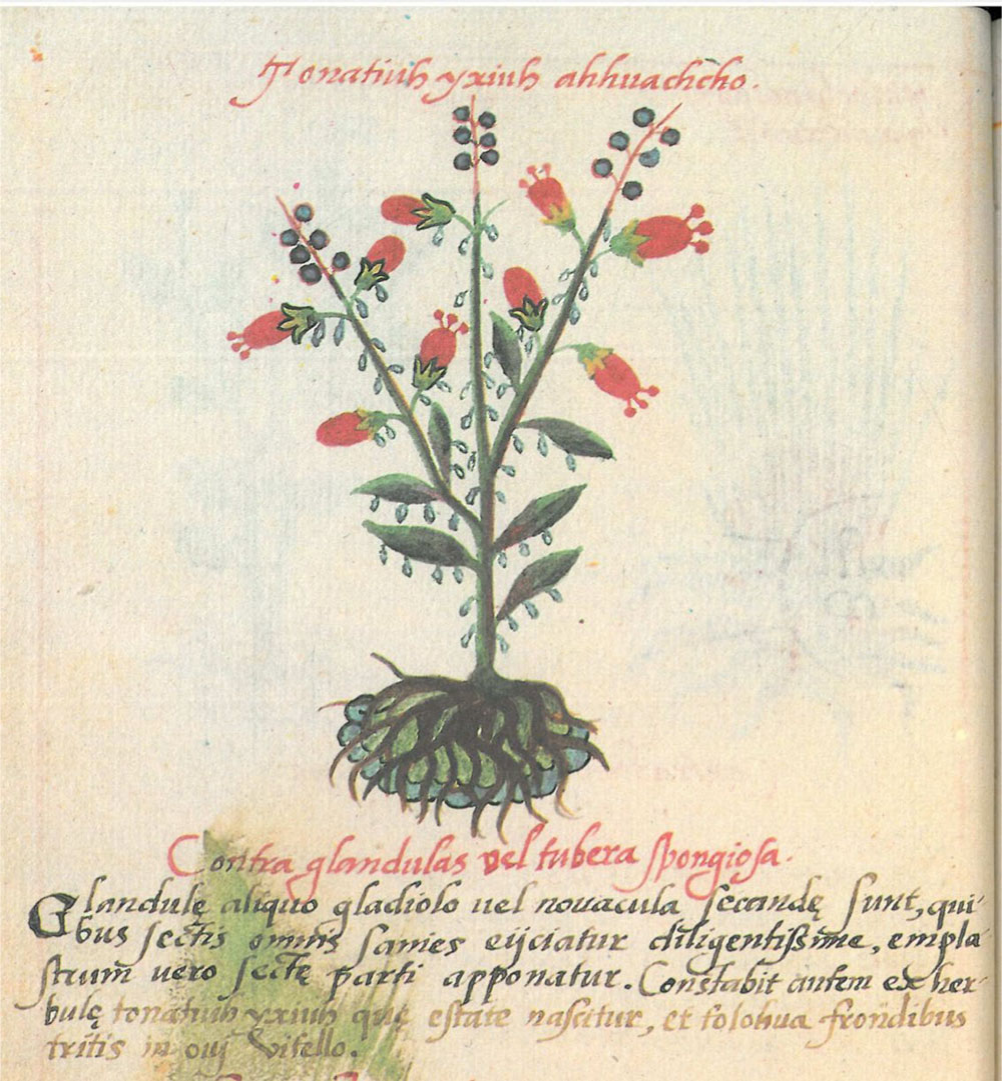
Cruz‐Badianus codex, 28v.

Because of its extensive description of plants and their medicinal uses as well as detailed pictures, the codex has attracted interest in the pharmacological properties of the plants (see Chavarría & Espinosa, 2019 for review). For example, for glandular swellings, the manuscript recommends that “the tumors are to be cut with a small sword or knife, which done the matter is carefully cleared out, and a plaster applied to the cut” (Gates, 1939, 42). This plaster contains the plant *tonatiuh yxiuh* (Fig. 4), identified as the *Heimia salicifolia*, which has anti‐inflammatory, ataractic (soothing), and antispasmodic properties (Malone & Rother, 1994), and crushed leaves of the *tolohua* (*Datura* spp. (Solanacea)), which is a powerful narcotic and analgesic (Geck, Lecca, Marchese, Casu, & Leonti, 2021). The dressing would have reduced inflammation and pain following the surgical procedure.

Overall, we see that the Mesoamerican cosmovision guides the use of plants for medicinal purposes. The association of body and cosmos, the tripartite distinction of souls, and the balancing of hot and cold are fundamental elements of the Cruz‐Badianus codex. Contemporary Nahua of the Sierra de Zongolica still have an extensive botanical knowledge. Weimann and Heinrich (1997) documented the use of 203 plants in this region for a variety of uses, such as dermatological problems, respiratory illnesses, and gynecological issues.

Researchers with pharmacological interests tend to focus on the purely chemical properties of plants, and whether they are useful in a western medical setting (e.g., Chavarría & Espinosa, 2019). We hypothesize that the philosophical ideas that underlie the medicinal uses prescribed in the codex are not incidental, but rather help to scaffold knowledge, retain in memory successful remedies, and aid the transmission of information. For example, some plants in the Mesoamerican cosmovision are associated with cold and are used to treat diseases that are described as hot (García‐Hernández, Vargas‐Guadarrama, & Vibrans, 2023). Rather than the real properties of plants (as shown, for example, by their temperature), these notions of hot and cold are conceptual. A fever or overheated eyes—conceived of as hot—is combated with plants that fight the heat, such as *oco‐xochitl*, *huacal‐xochitl*, *matlal‐xochitl*, and *tlaco‐izqui‐xochitl—*conceptualized as cold, and thus as restoring the body's equilibrium. The reverse also holds: a cold affliction is fought with a hot remedy to redress the body's balance. Cold and heat in a humoral system are thus not physiological but conceptual; the hot‐cold system is a cognitive technology that helps retain in memory which remedies go with which diseases. In the next two sections, we will look in more detail at attempts to isolate the efficacious parts of Indigenous science from the broader philosophical foundations, how this fits in discussions on colonization and decolonization, and the use of cognitive technologies.

## 4 Native science and European science

In a foundational study of the cognitive basis of biology, Brent Berlin (1992) hypothesized that folk biological taxonomic classifications have strong cross‐cultural similarities (especially at the species level) because we naturally and instinctively apprehend the natural world as it is. Humans are “drawn by some kind of innate curiosity to those groupings of plants and animals that represent the most distinctive chunks of biological reality” (Berlin, 1992, 290). Berlin (1973) also drew a connection between Linnaeus’ taxonomy and folk taxonomies across the world. Each classification has a fixed rank (e.g., genus, species) and a specific content (e.g., carnivora, brown bear): “Linnaeus and his predecessors formally codified a system of nomenclature present in the folk systematics of earliest prescientific man and still recognized in the natural folk biological systems of classification found in the languages of preliterate peoples today” (Berlin, 1973, 265).

However, critics such as the cultural anthropologist Scott Atran (1993) have pointed out that conceptual processing is a much more active process, which requires attention for morphological features, ecological salience, and usefulness for humans. The sophisticated pharmacological knowledge contained in the Cruz‐Badianus codex is not the result of mere passive curiosity. Rather, this knowledge likely involved trial and error, cumulative retention of successful interventions, analogical reasoning, and the use of conceptual schemas (such as the cold‐hot humoral classification) to aid memorization. The taxonomic classification reflects cultural salience, and Indigenous folk systems organize different kinds of plants “in ways that assist memories and allow communication about species that are important in particular cultural contexts (including world views, practices, and culturally important species). They are therefore more variable in structure and generally less extensive than scientific taxonomies” (Turner, Burton, & Van Eijk, 2013, 151). Some such conceptual schemas we have identified as part of the Mesoamerican cosmovision.

To understand the influence of the Cruz‐Badianus codex on western medicine and knowledge of plants, it is important to challenge the eurocentrism that still pervades a lot of the history of science. According to the standard view, science is an exclusively western invention, and any influence goes from western to nonwestern cultures. However, recent analyses, among others by the historian of science James Poskett (2022), show a bidirectional influence. By the mid to late 16th century, American plants slowly made their way into European pharmacies and kitchens. The Cruz‐Badianus codex describes and illustrates several of them, including cacao and vanilla. In the early 17th century, the codex came into the custody of Diego Cortavila y Sanabria, apothecary of the Spanish king Felipe IV, who studied it for its pharmacological properties. Around 1625, it was obtained by cardinal Francisco Barberini and subsequently entombed in the Barberini library for the next three centuries.

Remedies from the Cruz‐Badianus codex and other early Mesoamerican sources made their way into Spanish pharmacopoeia. If the remedies worked, they were used. Renaissance Spain had at this point already absorbed significant medicinal knowledge from the Islamic world, part of which it had conquered during the Reconquista period. This had led to an openness to a variety of different sources of knowledge and practices, including in the medical domain (Waldstein, 2014). The Franciscan friar Bernardino de Sahagún supervised an ethnographic and natural historical study of Mesoamerica (original title: *La Historia General de las Cosas de Nueva España*), in which he translated and edited Nahuatl texts. This is now known as the Florentine codex, composed in the second half of the 16th century. This codex was made in partnership with Nahua elders and former students of the earlier‐mentioned Colegio de Santa Cruz. It contains a wealth of information on Indigenous philosophical views and religious beliefs and practices, as well as societal structures, economy, and knowledge of the local fauna and flora.^4^ The compilation of this codex and the initial reception of the Cruz‐Badianus manuscript indicate that European audiences were curious about and open to Native societies and their cultures. This went beyond the evangelizing goals that New Spanish (colonial) authorities clearly had in mind.

Only recently, historians of science and of philosophy are appreciating the profound influence of Indigenous American customs, plant taxonomies, and philosophies on western culture (see, e.g., Park, 2013). As European scholars relied on the findings and observations of Indigenous people to enlarge their biological and other knowledge, they realized that the worldview they had inherited from authors from antiquity and the Bible was incomplete. Scientific classification such as the Linnean system reflected European folk classifications (Berlin, 1973) and could not easily incorporate all these new plants, as well as the increasing number of fossils that were being unearthed. Eventually, these difficulties contributed to a profound reconceptualization of the natural world as not static and fixed, but in constant flux, changing western science, eventually leading to proto‐evolutionary ideas voiced by, among others, Buffon (1766) and Treviranus (1805).

The influx of Mesoamerican knowledge of plants and its influence on early modern science is illustrative of how humans learn more generally. Early in development, violations of core knowledge make infants attentive and curious, and prompt them to investigate. As Stahl and Feigenson (2019, 136) hypothesize, “when predictions generated by core knowledge fail to match the observed data, infants and children experience an enhanced drive to seek and retain new information.” The theory‐like character of core knowledge leads us to expect similarities in how children and scientists learn (Carey & Spelke, 1996).

As Adam Smith already observed in his *History of Astronomy* (1795), when we detect anomalies in our theories, we are prompted to re‐examine them, possibly leading to deeper insights. A naturalist gazes at “a singular plant, or a singular fossil, that is presented to him” (Smith, 1795, 10) and that does not fit his classification scheme. This anomalous plant or fossil thus becomes a source of wonder. Smith hypothesized that the cognitive emotions of surprise and wonder make us attentive to gaps in our knowledge, and that this contributes to changes in scientific theories (see De Cruz, 2024, chapter 2). He thereby anticipated later authors such as Thomas Kuhn (1962) who emphasize the importance of anomalies for scientific thinking.

## 5 Decolonizing Indigenous cognitive technologies

An estimated 80% of our current drugs are based on plants (Bauer & Brönstrup, 2014). A lot of this knowledge originally comes from Indigenous sources. Because of the great biodiversity in areas managed by Indigenous people, many traditional remedies continue to show potential for pharmacological uptake. For example, Molimau‐Samasoni, Woolner, Foliga, Robichon, and Patel (2021) studied the bioactive components of *Psychotria insularum*, a plant widely used in Samoan traditional medicine to treat inflammation associated with fever, body aches, swellings and wounds, and found pharmacological potential. Similar successful studies have been conducted into the ethnobotanical knowledge of the Irulas from southern India (Ragupathy & Newmaster, 2009). As we have seen, Indigenous cognitive technologies help to retain and transmit this knowledge, which is culturally embedded and reflects centuries of natural experiments.

The concept of Indigenous science refers to the idea that Indigenous people have “their own systems of knowledge for observing, collecting, categorizing, recording, using, disseminating and revising information and concepts that explain how the world works; they use their own knowledge systems to ensure the flourishing of their communities’ health, livelihood, vibrancy and self‐determination” (Whyte, Brewer, & Johnson, 2016, 25–26). However, the pharmacological testing of Indigenous botanical knowledge divorces it from its original context. Often the people who originally came up with the remedies are unable to access the expensive medicine based on it, and rarely do patents give credit to Indigenous communities, a practice that is referred to as “biopiracy” (Imran, Wijekoon, Gonawala, Chiang, & De Silva, 2021).

These malpractices follow colonial‐era views where Indigenous (and more broadly nonwestern) science and philosophy were seen as not real science or philosophy (see Sanchez‐Perez, 2024 for discussion). Yet, medicinal plants are harvested for use in western science and enterprises, which benefit from centuries of Indigenous experiments and cognitive technologies. In this context, decolonizing would require open and transparent databases that can also be consulted by local people, and acknowledgment of Indigenous medical practitioners not only in the initial (publicly funded) papers that appear on these topics, but also in the patents of medication that is developed on the basis of their knowledge. Ideally, some of the profits that pharmaceutical companies make would go to Indigenous communities.

Crucial elements of increasing Indigenous people's participation in leadership include involving them in decision‐making and disseminating tools to better help survey the natural resources they manage. We provide two examples of initiatives that could be applied to pharmaceutical plant management.

The United Nations Development Program^5^ has outlined recommendations for how Indigenous communities can be included in leadership to adapt to and mitigate climate change, but a lot of their recommendations are also useful to consider the pharmacological use of plants. Indigenous people steward about 25% of the world's land and about 36% of intact forest. This stewardship helps protect biodiversity and can aid the absorption of CO₂. In addition, these areas are also huge sources of plants with potentially medicinal uses. Indigenous people thus provide a service to the rest of the world, particularly as modern medicine continues to depend on traditional plant sources. With the spread of novel and older infectious diseases due to climate change and migration (and tourism) across the globe, and the increase in cancers and other diseases, it is vital that we continue to have access to these resources. To help Indigenous people do this, and to give them due credit, they should not only be acknowledged as contributors to cutting‐edge scientific knowledge, but also be involved in decision‐making, notably about the future of the often vulnerable landscapes they manage and about how their pharmaceutical knowledge is put to use in labs and universities. Agencies such as the United Nations’ Local Communities and Indigenous Peoples Platform^6^ which is currently involved with climate change and Indigenous knowledge could likewise work to ensure the integration of Indigenous pharmaceutical knowledge and practices into international and national programs and policies.

To be able to participate in land management, use of relevant software such as for geospatial services and training is crucial. The anthropologist Thomas Moore advocates working with existing Indigenous people's organizations, rather than try and create new structures from scratch. These organizations can then learn how to use databases and other tools free of charge, as in a recent initiative to include Indigenous people from Amazonia in geospatial services (Moore, 2022).

## 6 Conclusion

We have examined the role of cognitive technologies in the transmission and construction of Mesoamerican botanical knowledge in the Cruz‐Badianus codex, a medicinal herbarium from the 16th century. As we showed, the Mesoamerican cosmovision was instrumental in how Nahua in this period thought about human health, and how plants could contribute to it. While we have cognitive dispositions that help us to learn about botany, the culture‐specific concepts embedded in the Mesoamerican cosmovision helped both memorization and transmission of ethnobotanical knowledge. We have shown how this Indigenous knowledge had an influence on early modern science, by challenging classification schemes and ideas about botany. Indigenous cognitive technologies have contributed to and vastly enlarged western medicine, but this has gone largely unacknowledged.

## Supporting information


Supporting Information

## Notes

1 Nahuatl is a group of languages of the Uto‐Aztecan language family, one of the language groups still spoken by Indigenous Mexican people today.

2 All translations are by William Gates (1939, reprinted 2000).

3 It is revealing to see differences in healing practices between a living tradition (Qollahuaya) versus a tradition we know only from written testimony (Nahua). Next to healers, the Qollahuaya also have diviners and other specialists who perform rituals to restore the balance between the mountain and the deities that inhabit it and patients with difficult‐to‐cure diseases that may require antibiotics such as tuberculosis (see Bastien, 1985, 600−601 for an extensive description of such a ritual for renal tuberculosis). It seems not unlikely that such interventions also took place in precolonial Mexico, but the Nahua manuscript lacks information about such rituals: even though it is written and illustrated by knowledgeable contemporaries, it only describes medical interventions with plants and minerals, strictly without supernatural help of deities that the Christian colonizers would have rejected. After all, as recent converts, the authors had an interest in concealing (or perhaps suppressing) divination and appeals to supernatural interventions that were not directed at the Christian god. Perhaps for a similar reason, the manuscript does not mention music therapy, which the Qollahuaya also use, for instance, the playing of *qantu* panpipes seeks to alleviate depression, melancholy, sadness, and sorrow (Hachmeyer, 2019).

4 One can consult a digital project that gives a good overview of the Florentine codex at: https://florentinecodex.getty.edu/

5
https://social.desa.un.org/issues/indigenous‐peoples/indigenous‐peoples‐at‐the‐united‐nations

6
https://lcipp.unfccc.int/homepage

## References

[tops70008-bib-0001] Atran, S. (1993). Folk biological cognition. Current Anthropology, 34(2), 195–198.

[tops70008-bib-0002] Atran, S. (1999). Itzaj Maya folkbiological taxonomy: Cognitive universals and cultural particulars. In D. L. Medin & S. Atran (Eds.), Folkbiology (pp. 119–203). Cambridge, MA: MIT Press.

[tops70008-bib-0003] Bacon, B. , Khatiri, A. , Palmer, J. , Freeth, T. , Pettitt, P. , & Kentridge, R. (2023). An Upper Palaeolithic proto‐writing system and phenological calendar. Cambridge Archaeological Journal, 33(3), 371–389.

[tops70008-bib-0004] Baillargeon, R. (1987). Object permanence in 3½‐ and 4½‐month‐old infants. Developmental Psychology, 23(5), 655–664.

[tops70008-bib-0005] Barwick, L. (2023). Songs and the deep present. In A. McGrath , L. Rademaker , & J. Troy (Eds.), Everywhen: Australia and the language of deep history (pp. 93–122). Lincoln: University of Nebraska Press.

[tops70008-bib-0006] Bastien, J. W. (1978). Mountain/body metaphor in the Andes. Bulletin de l'Institut Français d'Études Andines, 7(1–2), 87–103.

[tops70008-bib-0007] Bastien, J. W. (1982). Herbal curing by Qollahuaya Andeans. Journal of Ethnopharmacology, 6, 13–28. 7109663 10.1016/0378-8741(82)90069-1

[tops70008-bib-0008] Bastien, J. W. (1985). Qollahuaya‐Andean body concepts: A topographical‐hydraulic model of physiology. American Anthropologist, 87(3), 595–611.

[tops70008-bib-0009] Berlin, B. (1973). Folk systematics in relation to biological classification and nomenclature. Annual Review of Ecology and Systematics, 4, 259–271.

[tops70008-bib-0010] Berlin, B. (1992). Ethnobiological classification: Principles of categorization of plants and animals in traditional societies. Princeton, NJ: Princeton University Press.

[tops70008-bib-0011] Bender, A. , & Beller, S. (2011). The cultural constitution of cognition: Taking the anthropological perspective. Frontiers in Psychology, 2, 67. 21716578 10.3389/fpsyg.2011.00067 PMC3110796

[tops70008-bib-0012] Bauer, A. , & Brönstrup, M. (2014). Industrial natural product chemistry for drug discovery and development. Natural Product Reports, 31(1), 35–60. 24142193 10.1039/c3np70058e

[tops70008-bib-0013] de Buffon, C. (G.‐L. Leclerc) (1766). Histoire naturelle, générale et particulière. Vol. 14. Paris: Imprimerie Royale.

[tops70008-bib-0014] Cardoso, D. B. , de Queiroz, L. P. , Bandeira, F. P. , & Góes‐Neto, A. (2010). Correlations between indigenous Brazilian folk classifications of fungi and their systematics. Journal of Ethnobiology, 30(2), 252–264.

[tops70008-bib-0015] Carey, S. , & Spelke, E. (1996). Science and core knowledge. Philosophy of Science, 63(4), 515–533.

[tops70008-bib-0016] Chavarría, A. , & Espinosa, G. (2019). Cruz‐Badiano codex and the importance of the Mexican medicinal plants. Journal of Pharmaceutical Technology, Research and Management, 7(1), 15–22.

[tops70008-bib-0017] De Cruz, H. , & De Smedt, J. (2007). The role of intuitive ontologies in scientific understanding–The case of human evolution. Biology & Philosophy, 22, 351–368.

[tops70008-bib-0018] De Cruz, H. , & De Smedt, J. (2022). Melioristic genealogies and Indigenous philosophies. Philosophical Forum, 53(4), 209–226.

[tops70008-bib-0019] De Cruz, H. (2024). Wonderstruck: How wonder and awe shape the way we think. Princeton, NJ: Princeton University Press.

[tops70008-bib-0020] de la Cruz, M. (1552). (trans: J. Badianus ). Libellus de Medicinalibus Indorum Herbis . Retrieved from https://commons.wikimedia.org/wiki/File:LIbellus_de_Medicinalibus_Indorum_Herbis.pdf

[tops70008-bib-0021] De Smedt, J. , & De Cruz, H. (2011). The role of material culture in human time representation: Calendrical systems as extensions of mental time travel. Adaptive Behavior, 19(1), 63–76.

[tops70008-bib-0022] De Smedt, J. , & De Cruz, H. (2023). Animisms: Practical Indigenous philosophies. In T. Smith (Ed.), Animism and philosophy of religion (pp. 95–122). Basingstoke: Palgrave.

[tops70008-bib-0023] Diamond, J. , & Bishop, K. D. (1999). Ethno‐ornithology of the Ketengban people, Indonesian New Guinea. In D. L. Medin & S. Atran (Eds.), Folkbiology (pp. 17–45). Cambridge, MA: MIT Press.

[tops70008-bib-0024] Donald, M. (1991). Origins of the modern mind: Three stages in the evolution of culture and cognition. Cambridge, MA: Harvard University Press.

[tops70008-bib-0025] Foster, G. M. (1987). On the origin of humoral medicine in Latin America. Medical Anthropology Quarterly, 1(4), 355–393.

[tops70008-bib-0026] Frank, E. T. , Buffat, D. , Liberti, J. , Aibekova, L. , Economo, E. P. , & Keller, L. (2024). Wound‐dependent leg amputations to combat infections in an ant society. Current Biology, 34(14), 3273–3278. 38959879 10.1016/j.cub.2024.06.021

[tops70008-bib-0027] Freymann, E. , Carvalho, S. , Garbe, L. A. , Ghazhelia, D. D. , Hobaiter, C. , Huffman, M. A. , Muhumuza, G. , Schulz, L. , Sempebwa, D. , Wald, F. , Yikii, E. R. , Zuberbühler, K. , & Schultz, F. (2024). Pharmacological and behavioral investigation of putative self‐medicative plants in Budongo chimpanzee diets. PLOS One, 19(6), e0305219. 38900778 10.1371/journal.pone.0305219 PMC11189245

[tops70008-bib-0028] Furst, J. (1995). A natural history of the soul in ancient Mexico. New Haven, CT: Yale University Press.

[tops70008-bib-0029] García‐Hernández, K. Y. , Vibrans, H. , Colunga‐García Marín, P. , Vargas‐Guadarrama, L. A. , Soto‐Hernández, M. , Katz, E. , & Luna‐Cavazos, M. (2021). Climate and categories: Two key elements for understanding the Mesoamerican hot‐cold classification of illnesses and medicinal plants. Journal of Ethnopharmacology, 266, 113419. 33002566 10.1016/j.jep.2020.113419

[tops70008-bib-0030] García‐Hernández, K. Y. , Vargas‐Guadarrama, L. A. , & Vibrans, H. (2023). Academic history, domains and distribution of the hot‐cold system in Mexico. Journal of Ethnobiology and Ethnomedicine, 19(1), 50. 37919763 10.1186/s13002-023-00624-1 PMC10623800

[tops70008-bib-0031] Gates, W. (1939) (reprinted 2000). The De la Cruz‐Badiano Aztec herbal of 1552. Mineola, NY: Dover.

[tops70008-bib-0032] Geck, M. S. , Lecca, D. , Marchese, G. , Casu, L. , & Leonti, M. (2021). Ethnomedicine and neuropsychopharmacology in Mesoamerica. Journal of Ethnopharmacology, 278, 114243. 34129899 10.1016/j.jep.2021.114243

[tops70008-bib-0033] Gibson, J. J. (1979). The ecological approach to visual perception. Boston, MA: Houghton Mifflin.

[tops70008-bib-0034] Gimmel, M. (2008). Reading medicine in the Codex de la Cruz Badiano. Journal of the History of Ideas, 69(2), 169–192. 19127831 10.1353/jhi.2008.0017

[tops70008-bib-0035] Gladwin, T. (1970). East is a big bird. Navigation and logic on Puluwat atoll. Cambridge, MA: Harvard University Press.

[tops70008-bib-0036] Hachmeyer, S. (2019). The uncontrolled equivocation of music therapy: Health, illness and musical healing among the Kallawaya in the northern Bolivian Andes. Musicology Research Journal, 6, 51–82.

[tops70008-bib-0037] Hassig, D. (1989). Transplanted medicine: Colonial Mexican herbals of the sixteenth century. RES: Anthropology and Aesthetics, 17(1), 30–53.

[tops70008-bib-0038] Imran, Y. , Wijekoon, N. , Gonawala, L. , Chiang, Y. C. , & De Silva, K. R. D. (2021). Biopiracy: Abolish corporate hijacking of Indigenous medicinal entities. Scientific World Journal, 2021(1), 8898842. 33679261 10.1155/2021/8898842 PMC7910072

[tops70008-bib-0039] Izard, V. , Dehaene‐Lambertz, G. , & Dehaene, S. (2008). Distinct cerebral pathways for object identity and number in human infants. PLoS Biology, 6(2), e11. 18254657 10.1371/journal.pbio.0060011 PMC2225438

[tops70008-bib-0040] Kovács, Á. M. , Téglás, E. , & Endress, A. D. (2010). The social sense: Susceptibility to others’ beliefs in human infants and adults. Science, 330(6012), 1830–1834. 21205671 10.1126/science.1190792

[tops70008-bib-0041] Kuhn, T. S. (1962). The structure of scientific revolutions. Chicago, IL: Chicago University Press.

[tops70008-bib-0042] LaRochelle, S. , & Berkes, F. (2003). Traditional ecological knowledge and practice for edible wild plants: Biodiversity use by the Rarámuri, in the Sierra Tarahumara, Mexico. International Journal of Sustainable Development & World Ecology, 10(4), 361–375.

[tops70008-bib-0043] Laumer, I. B. , Rahman, A. , Rahmaeti, T. , Azhari, U. , Hermansyah , Atmoko, S. S. U. , & Schuppli, C. (2024). Active self‐treatment of a facial wound with a biologically active plant by a male Sumatran orangutan. Scientific Reports, 14(1), 8932. 38698007 10.1038/s41598-024-58988-7 PMC11066025

[tops70008-bib-0044] López Austin, A. (1996). La cosmovisión mesoamericana. In S. Lombardo & E. Nalda (Eds.), Temas mesoamericanos (pp. 471–507). Ciudad de México: Instituto Nacional de Antropología e Historia: Dirección General de Publicaciones, Consejo Nacional para la Cultura y las Artes.

[tops70008-bib-0045] Malone, M. H. , & Rother, A. (1994). *Heimia salicifolia*: A phytochemical and phytopharmacologic review. Journal of Ethnopharmacology, 42(3), 135–159. 7934084 10.1016/0378-8741(94)90080-9

[tops70008-bib-0046] Mansouri, I. , Ousaaid, D. , Squalli, W. , Douini, I. , Mounir, M. , El Agy, A. , Assouguema, A. , Achiband, H. , El Ghadraoui, L. , & Dakkic, M. (2021). Nest building, dimension, and selection of aromatic and medicinal twigs to repel ectoparasites in the European turtle dove. Journal of Animal Behaviour and Biometeorology, 9(4), 2133–2133.

[tops70008-bib-0047] Medin, D. L., & Atran, S. (1999). Folkbiology. Cambridge, MA: MIT Press.

[tops70008-bib-0048] Molimau‐Samasoni, S. , Woolner, V. H. , Foliga, S. E. T. , Robichon, K. , Patel, V. , Andreassend, S. K. , Sheridan, J. P. , Te Kawa, T. , Gresham, D. , Miller, D. , Sinclair, D. J. , La Flamme, A. C. , Melnik, A. V. , Aron, A. , Dorrestein, P. C. , Atkinson, P. H. , Keyzers, R. A. , Munkacsi, A. B. (2021). Functional genomics and metabolomics advance the ethnobotany of the Samoan traditional medicine “matalafi”. Proceedings of the National Academy of Sciences, 118(45), e2100880118. 10.1073/pnas.2100880118 PMC8609454 34725148

[tops70008-bib-0049] Moore, T. (2022). Including Indigenous peoples in geospatial services. Frontiers in Climate, 4, 810428.

[tops70008-bib-0050] Muñoz, S. J. C. (2017). El núcleo duro mesoamericano.¿Entre la unidad y la diversidad? Imago crítica. Revista de Antropología, Comunicación y Estudios Culturales, 6, 137–182.

[tops70008-bib-0052] Park, P. (2013). Africa, Asia, and the history of philosophy: Racism in the formation of the philosophical canon, 1780–1830. Albany, NY: SUNY Press.

[tops70008-bib-0053] Poskett, J. (2022). Horizons: A global history of science. London: Penguin.

[tops70008-bib-0054] Poole, S. (1981). Church law on the ordination of Indians and castas in New Spain. Hispanic American Historical Review, 61(4), 637–650.

[tops70008-bib-0055] Ragupathy, S. , & Newmaster, S. G. (2009). Valorizing the “Irulas” traditional knowledge of medicinal plants in the Kodiakkarai Reserve Forest, India. Journal of Ethnobiology and Ethnomedicine, 5, 10. 19366462 10.1186/1746-4269-5-10 PMC2681454

[tops70008-bib-0056] Robles‐Zamora, J. A. (2021). Morality as cognitive scaffolding in the nucleus of the Mesoamerican cosmovision. In J. De Smedt & H. De Cruz (Eds.), Empirically engaged evolutionary ethics (pp. 203–220). Cham: Springer (Synthese Library).

[tops70008-bib-0057] Salmón, E. (2000). Kincentric ecology: Indigenous perceptions of the human−nature relationship. Ecological Applications, 10(5), 1327–1332.

[tops70008-bib-0058] Salmón, E. (2020). Iwígara: The kinship of plants and people. American Indian ethnobotanical traditions and science. Portland, OR: Timber Press.

[tops70008-bib-0059] Sanchez‐Perez, J. (2024). Beyond gatekeeping: Philosophical sources, Indigenous philosophy, and the Huarochirí Manuscript. Metaphilosophy, 55(3), 365–380.

[tops70008-bib-0060] Schuppli, C. , & van Schaik, C. P. (2019). Animal cultures: How we've only seen the tip of the iceberg. Evolutionary Human Sciences, 1, e2. 37588402 10.1017/ehs.2019.1 PMC10427297

[tops70008-bib-0061] Smith, A. (1795). The history of astronomy. In J. Black & J. Hutton (Eds.), Essays on philosophical subjects (pp. 1–93). London: Cadell, Davies, and Creech.

[tops70008-bib-0062] Spelke, E. S. , & Kinzler, K. D. (2007). Core knowledge. Developmental Science, 10(1), 89–96. 17181705 10.1111/j.1467-7687.2007.00569.x

[tops70008-bib-0063] Stahl, A. E. , & Feigenson, L. (2019). Violations of core knowledge shape early learning. Topics in Cognitive Science, 11(1), 136–153. 30369059 10.1111/tops.12389 PMC6360129

[tops70008-bib-0064] Tewksbury, J. J. , Nabhan, G. P. , Norman, D. , Suzán, H. , Tuxill, J. , & Donovan, J. (1999). In situ conservation of wild chiles and their biotic associates. Conservation Biology, 13(1), 98–107.

[tops70008-bib-0065] Treviranus, G. R. (1805). Biologie, oder Philosophie der lebenden Natur für Naturforscher und Aerzte, Vol. 3. Göttingen: Johann Friedrich Röwer.

[tops70008-bib-0066] Tucker, A. O. , & Janick, J. (2020). Flora of the Codex Cruz‐Badianus. Cham: Springer.

[tops70008-bib-0067] Turner, N. J. , Burton, C. , & Van Eijk, J. (2013). Plants in language and classification among BC First Nations. BC Studies: The British Columbian Quarterly, 179, 135–158.

[tops70008-bib-0068] Waldstein, A. (2014). How can ethnobotany contribute to the history of Western herbal medicine? A Mesoamerican answer. In S. Francia & A. Stobart (Eds.), Critical approaches to the history of western herbal medicine (pp. 271–287). London: Bloomsbury.

[tops70008-bib-0069] Weimann, C. , & Heinrich, M. (1997). Indigenous medicinal plants in Mexico: The example of the Nahua (Sierra de Zongolica). Botanica Acta, 110(1), 62–72.

[tops70008-bib-0070] White, L. (1967). The historical roots of our ecologic crisis. Science, 155(3767), 1203–1207. 17847526 10.1126/science.155.3767.1203

[tops70008-bib-0071] Whyte, K. P. , Brewer, J. P. , & Johnson, J. T. (2016). Weaving Indigenous science, protocols and sustainability science. Sustainability Science, 11, 25–32.

[tops70008-bib-0072] Whiten, A. , Goodall, J. , McGrew, W. C. , Nishida, T. , Reynolds, V. , Sugiyama, Y. , Tutin, C. E. , Wrangham, R. W. , Boesch, C. (1999). Cultures in chimpanzees. Nature, 399(6737), 682–685. 10385119 10.1038/21415

[tops70008-bib-0051] Yinda, L. E. D. O. , Onanga, R. , Obiang, C. S. , Begouabe, H. , Akomo‐Okoue, E. F. , Obame‐Nkoghe, J. , Mitola, R. , Ondo, J.‐P. , Atome, G.‐R. N. , Obame Engonga , L.‐C. , Ibrahim , Setchell, J. M. , Godreuil, S. (2024). Antibacterial and antioxidant activities of plants consumed by western lowland gorilla (*Gorilla gorilla gorilla*) in Gabon. PLOS One, 19(9), e0306957. 39259705 10.1371/journal.pone.0306957 PMC11389915

