# Supplementary material for: Cosmovision as Cognitive Technology: The Case of Mesoamerican Medicinal Knowledge
Source: Top Cogn Sci. 2025 Apr 28;18(1):92–110. doi: 10.1111/tops.70008 (PMC12831609; doi:10.1111/tops.70008)
Supplement: Supplementary file 1 — Supporting Information [file TOPS-18-92-s001.docx]

**Supplementary materials:**

None

**Copyright of images:**

All imagery is taken from the creative common licensed Wikimedia Commons copy of the Cruz-Badianus codex, which can be found at <https://commons.wikimedia.org/wiki/File:Libellus_de_Medicinalibus_Indorum_Herbis.pdf>

**Photo release:**

Not applicable
